# Transit and integration of extracellular mitochondria in human heart cells

**DOI:** 10.1038/s41598-017-17813-0

**Published:** 2017-12-12

**Authors:** Douglas B. Cowan, Rouan Yao, Jerusha K. Thedsanamoorthy, David Zurakowski, Pedro J. del Nido, James D. McCully

**Affiliations:** 1 Department of Anesthesiology, Perioperative and Pain Medicine, Boston Children’s Hospital, Boston, 02115 Massachusetts USA; 2 Department of Anæsthesia, Harvard Medical School, Boston, 02115 Massachusetts USA; 3 Harvard Stem Cell Institute, Harvard University, Cambridge, 02138 Massachusetts USA; 4 Department of Cardiac Surgery, Boston Children’s Hospital, Boston, 02115 Massachusetts USA; 5 Department of Surgery, Harvard Medical School, Boston, 02115 Massachusetts USA

## Abstract

Tissue ischemia adversely affects the function of mitochondria, which results in impairment of oxidative phosphorylation and compromised recovery of the affected organ. The impact of ischemia on mitochondrial function has been extensively studied in the heart because of the morbidity and mortality associated with injury to this organ. As conventional methods to preserve cardiac cell viability and contractile function following ischemia are limited in their efficacy, we developed a unique approach to protect the heart by transplanting respiration-competent mitochondria to the injured region. Our previous animal experiments showed that transplantation of isolated mitochondria to ischemic heart tissue leads to decreases in cell death, increases in energy production, and improvements in contractile function. We also discovered that exogenously-derived mitochondria injected or perfused into ischemic hearts were rapidly internalised by cardiac cells. Here, we used three-dimensional super-resolution microscopy and transmission electron microscopy to determine the intracellular fate of endocytosed exogenous mitochondria in human iPS-derived cardiomyocytes and primary cardiac fibroblasts. We found isolated mitochondria are incorporated into cardiac cells within minutes and then transported to endosomes and lysosomes. The majority of exogenous mitochondria escape from these compartments and fuse with the endogenous mitochondrial network, while some of these organelles are degraded through hydrolysis.

## Introduction

Mitochondria play an essential role in energy production and cellular homeostasis. Dysfunction of these organelles as a result of ischemia or genetic mutations can lead to the loss of high-energy phosphate reserves, accumulation of mitochondrial calcium, and a buildup of reactive oxygen molecules^1–5^. Our previous studies demonstrated that transplantation of isolated mitochondria to the ischemic heart leads to reductions in infarct size, increases in adenosine triphosphate (ATP) production, and improvements in contractility^6,7^. We also observed that mitochondria injected or perfused into the heart were rapidly internalised by a variety of cardiac cells including cardiomyocytes and fibroblasts^7,8^. Additional experiments using cell cultures proved that the uptake of mitochondria occurs through actin-dependent endocytosis and results in rescue of cellular function by increasing energy production and replenishing mitochondrial DNA (mtDNA)^9^. Although other researchers have observed endocytic incorporation of extracellular mitochondria, the intracellular trafficking and fate of these organelles remains unknown^10–15^.

In this study, we used three-dimensional super-resolution structured illumination microscopy (3-D SR-SIM) and transmission electron microscopy (TEM) to reveal the intracellular position of endocytosed mitochondria in human induced pluripotent stem cell-derived cardiomyocytes (iPS-CMs) and human cardiac fibroblasts (HCFs). By labelling isolated mitochondria with fluorescent proteins or gold nanoparticles, we were able to observe the transit of exogenous mitochondria in these cells. Distinct fluorescent labelling of various cell compartments in iPS-CMs and HCFs allowed us to visualise the progression of exogenous mitochondria through the endolysosomal system and establish that these organelles primarily integrate with the endogenous mitochondrial network in both cardiac cell types. Immunoblot experiments confirmed that the cardiomyocytes and fibroblasts used in these studies expressed proteins compatible with mitochondrial fusion.

When combined with the findings of other investigators, our results strongly support the notion that the uptake and subsequent fusion of extracellular mitochondria with recipient cell mitochondria is an evolutionarily-conserved and pervasive biological process^7–16^. A thorough understanding of the endocytic uptake, intracellular transit, and mitochondrial integration of exogenous mitochondria in cells may present new treatment strategies for the ischemic heart and drive the development of organelle-based therapeutics for a host of other human diseases and disorders^17–20^.

## Results

### Labelling of organelles and characterisation of isolated mitochondria

**Figure 1:**
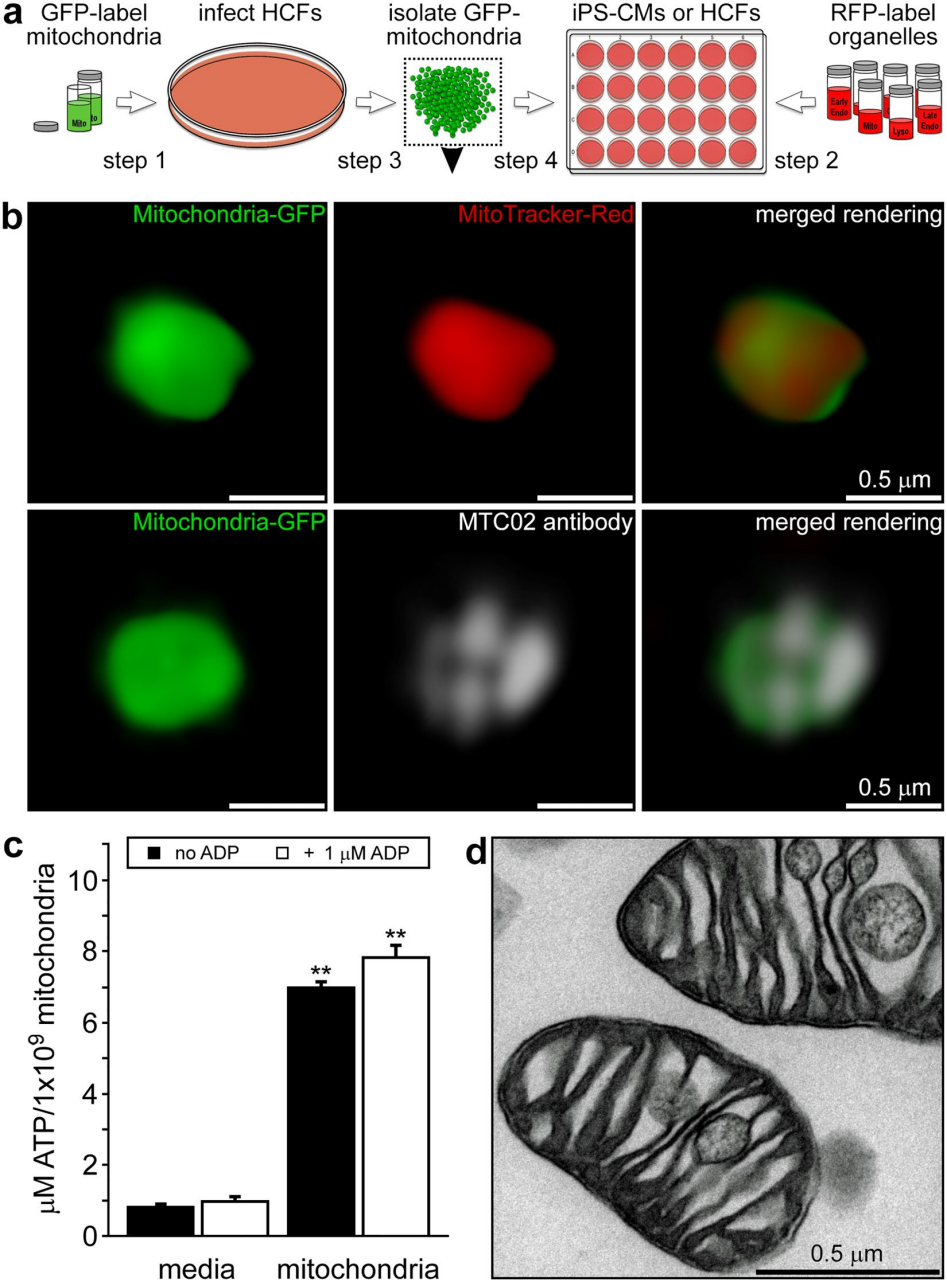
Experimental strategy and characterisation of isolated human fibroblast mitochondria. (**a)** HCFs infected with BacMam CellLight Mitochondria-GFP were used for mitochondrial isolations and iPS-CMs or HCFs on coverslips were infected with RFP CellLight reagents to label specific cell structures. Isolated GFP-labelled mitochondria were added to RFP-labelled cells for 0 to 4 h. (**b)** 3-D SR-SIM of isolated HCF mitochondria stained with MitoTracker Red CMXRos (top panels) or the human mitochondria-specific antibody MTC02 (bottom panels). Isolated mitochondria, stained mitochondria, and the combined image are shown left to right. Scale bars, 0.5 µm. (**c)** ATP content in media or isolated HCF mitochondria in the presence or absence of 1 µM ADP. Error bars indicate standard error of the mean. ATP levels were significantly higher in mitochondria than in media-only control groups (Student’s t-test, **p < 0.001). (**d)** Transmission electron microscopy of isolated HCF mitochondria. Scale bar, 0.5 µm. The image is representative of four separate mitochondrial isolations.

We investigated the temporal and spatial fate of endocytosed mitochondria in non-dividing iPS-CMs and dividing HCFs. The identity and morphology of these cardiac cells was substantiated by immunostaining with α-actinin (ACTN) and vimentin and both cell types were shown to react well with established mitochondrial antibodies (TOMM20 or MTC02) (Extended Data Fig. 1a). To discern exogenous mitochondria within cultured cells, we labelled HCF mitochondria with green fluorescent protein (GFP) and used red fluorescent proteins (RFP) to label various HCF and iPS-CM cell compartments through baculovirus-mediated transfer of mammalian fusion genes (Fig. 1a). Both cell types were readily infected with baculoviruses carrying fluorescent protein genes and exhibited specific expression of GFP or RFP in organelles including mitochondria, early and late endosomes, lysosomes, Golgi complexes, and the endoplasmic reticulum (Extended Data Fig. 1b). Isolated HCF GFP-labelled mitochondria were stained with MitoTracker Red CMXRos or a human mitochondria-specific antibody (MTC02) to confirm their identity and then imaged using 3-D SR-SIM (Fig. 1b). Isolated mitochondria were generally spherical in shape and varied in diameter from 250 to 2000 nm with the majority of these organelles falling within the 350 to 600 nm range^7^. In addition, adenosine triphosphate (ATP) measurements verified that isolated mitochondria were viable and energised (Fig. 1c), while transmission electron microscopy revealed these organelles were structurally intact (Fig. 1d)^7,8,21^.

### Endocytosis and intracellular position of exogenous mitochondria

**Figure 2:**
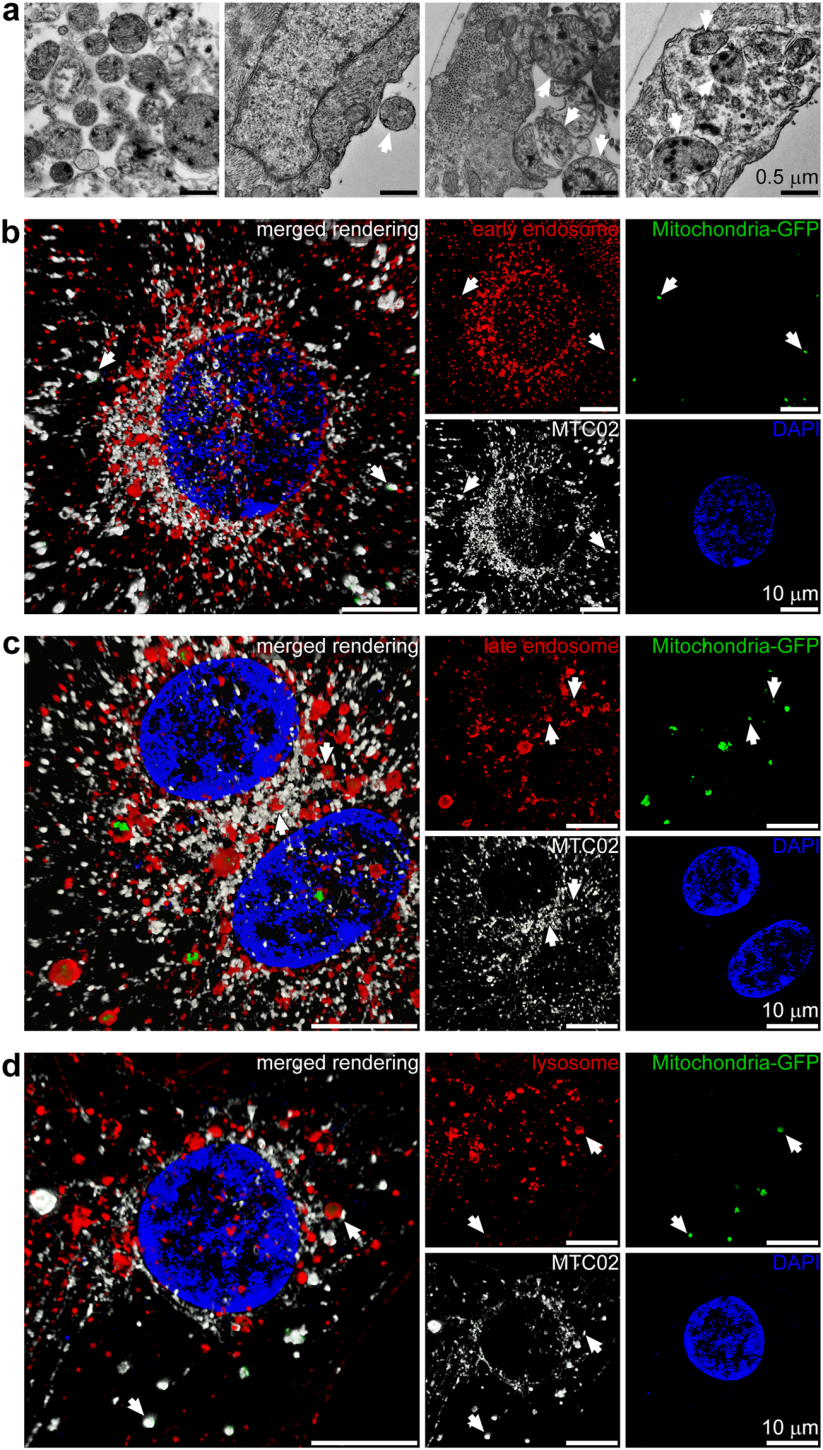
Uptake and endocytic transport of extracellular mitochondria in cardiomyocytes. (**a)** iPS-CMs were exposed to gold-labelled HCF mitochondria for 2.5, 5, and 10 min and imaged by TEM. Labelled mitochondria had electron dense deposits and were apparent at all three study times outside cells, adjacent to the apical cell surface, undergoing endocytosis, and inside cells (left to right and indicated by arrows). Images are representative of four experiments at 5 min. Scale bars, 0.5 µm. (**b)** Four color 3-D SR-SIM of colocalisation of exogenous mitochondria (green) with early endosomes (red) at 4 h. Nuclei were stained with DAPI (blue) and both endogenous and exogenous mitochondria were detected with the MTC02 antibody (white). The combined image (left) and each color (right) are shown. Scale bars, 10 µm. (**c)** Colocalisation of HCF mitochondria (green) with late endosomes (red) at 1 h. Scale bars, 10 µm. (**d)** Colocalisation of mitochondria (green) with lysosomes (red) at 4 h. Scale bars, 10 µm. In (**c**,**d)** arrows indicate examples of exogenous mitochondria associated with each compartment, which also reacted with the anti-mitochondria MTC02 antibody. Nuclei were stained with DAPI (blue) and images represent twelve separate volumes analysed at each time (0.5, 1, 2, and 4 h).

To examine endocytosis of isolated mitochondria in iPS-CMs, we first labeled HCF mitochondria with 10 nm diameter gold nanoparticles (Fig. 2a) to allow for their identification by transmission electron microscopy. Labelled exogenous mitochondria were observed outside cells, near cell surfaces, in the process of endocytic engulfment, and inside iPS-CMs^22^. Next, the intracellular position of endocytosed mitochondria in iPS-CMs was determined using four color 3-D SR-SIM (Fig. 2 and Extended Data Fig. 2). Because we did not observe GFP-labelled mitochondria associated with either the Golgi complex or endoplasmic reticulum, we focused on examining whether RFP-labelled endosomes, lysosomes, or endogenous mitochondria colocalised with internalised HCF mitochondria. Early endosomes (Fig. 2b), late endosomes (Fig. 2c), and lysosomes (Fig. 2d) of cardiomyocytes were all found to contain GFP-labelled mitochondria at each time examined (0.5, 1, 2, and 4 h). In addition, endocytosed mitochondria were found in close proximity to the endogenous mitochondrial network, which was generally located apical to the contractile apparatus (Extended Data Fig. 2a and Movie S1).

### Escape of exogenous mitochondria from the endolysosomal system

Exogenous mitochondria associated with early endosomes, late endosomes, and lysosomes were observed to be fully encapsulated or partly contained within these compartments. Mitochondria partially enclosed in endocytic vesicles were considered to be escaping from their respective endosomal or lysosomal compartments. Cell preparations were stained with anti-human mitochondria antibody (MTC02) to confirm that green fluorescence was attributable to the presence of intact organelles; though, this antibody was less reactive with encapsulated mitochondria—possibly a consequence of reduced antigen accessibility. To further corroborate the identity of the above compartments, we stained cardiomyocyte cell preparations with early endosome antigen 1 (EEA1) and the late endocytic marker Rab7, which helps control aggregation and fusion of late endosomes and lysosomes (Extended Data Fig. 2b–d)^23^. In addition, we observed some lysosomes associated with exogenous mitochondria stained for Rab7, which could simply epitomise the heterogeneity of the lysosomal compartment or indicate these particular compartments fused into endolysosomal vesicles^24,25^. We also noticed exogenous mitochondria in lysosomes that were Rab7 negative; however, we did not quantitate the proportion of internalised mitochondria in specific endocytic compartments expressing distinct protein compositions.

**Figure 3:**
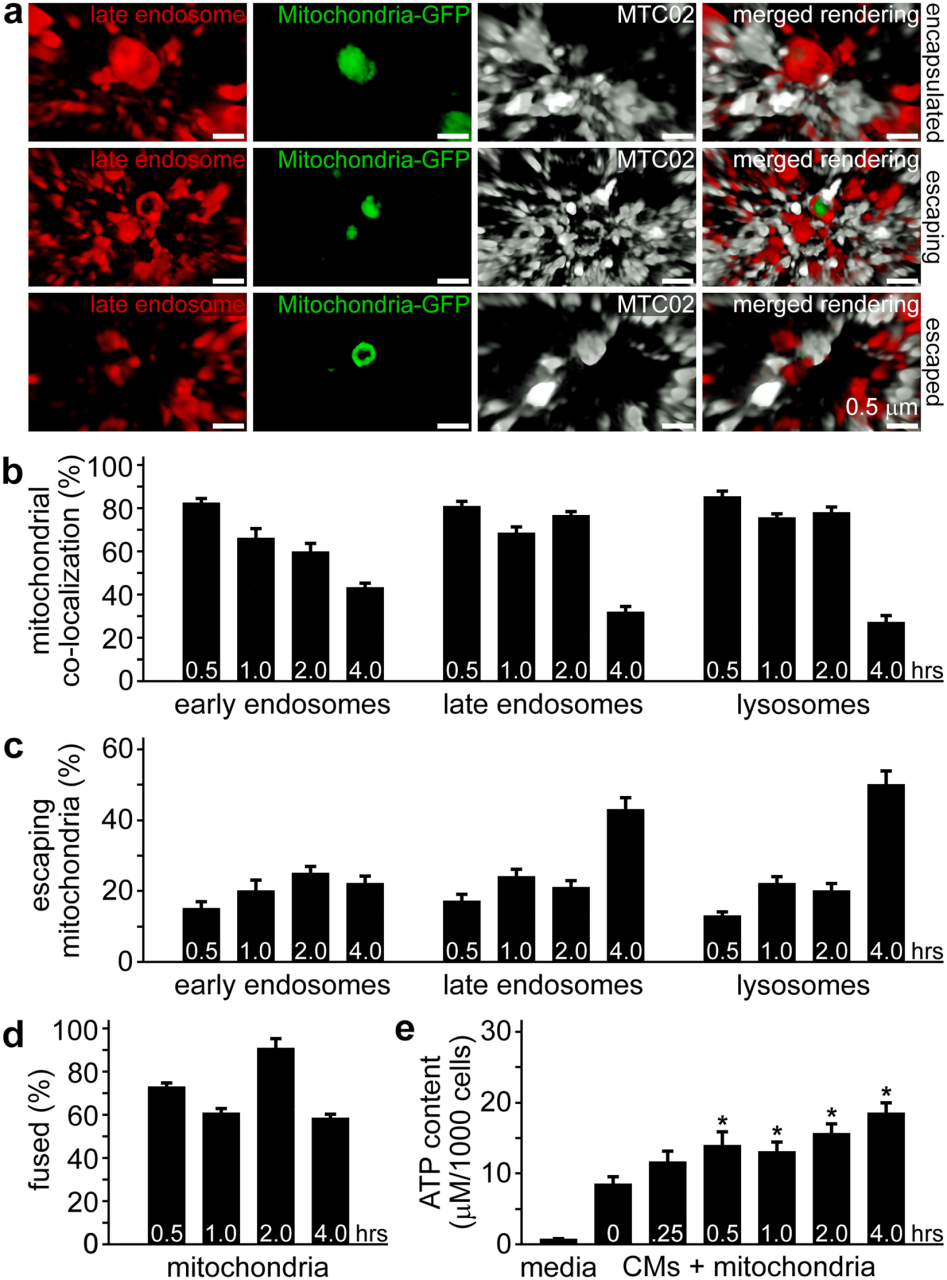
Encapsulation and escape of internalised mitochondria from cardiomyocyte cell compartments. (**a)** Late endosomes (red) associated with GFP-labelled exogenous mitochondria (green) were imaged by 3-D SR-SIM. Examples of encapsulated, escaping, and escaped mitochondria are shown after 1 h of incubation of isolated mitochondria and iPS-CMs. The anti-mitochondria antibody MTC02 demonstrated increased reactivity with exogenous mitochondria as they emerged from late endosomes and escaped mitochondria (bottom panels) become completely enveloped by MTC02. Images are representative of four experiments. Scale bars, 0.5 µm. (**b)** Quantitation of mitochondrial colocalisation with early endosomes, late endosomes, and lysosomes at 0.5, 1, 2, and 4 h. Results are expressed as the percentage of mitochondria associated with each compartment (*i.e*. encapsulated). (**c)** Quantitation of mitochondria escaping early endosomes, late endosomes, and lysosomes at 0.5, 1, 2, and 4 h. Results are expressed as the percentage of mitochondria associated with each compartment, but not fully encapsulated and represent a minimum of twelve experiments. (**d)** Quantitation of the colocalisation of endocytosed mitochondria with endogenous cardiomyocyte mitochondria. Results are expressed as the percentage of GFP-labelled mitochondria closely associated with RFP-labelled mitochondria. (**e)** ATP content of culture media and cardiomyocytes treated with isolated HCF mitochondria for 0, 0.25, 0.5, 1, 2, and 4 h. ATP content is expressed as µM per 1000 cells and error bars indicate standard error of the mean. ATP was significantly higher in iPS-CMs treated with mitochondria for 0.5, 1, 2, and 4 h compared to 0 h untreated cells (Student’s t-test, *p < 0.02). Results were obtained from twelve separate experiments.

The escape of exogenous mitochondria from endosomes and lysosomes was assessed by counting colocalisation of GFP-labelled mitochondria with each of these compartments. High-magnification 3-D SR-SIM suggested GFP-labelled mitochondria became increasingly reactive with MTC02 antibody upon emerging from late endosomes (Fig. 3a and Movie S2) as well as early endosomes and lysosomes (not shown). Enumeration of colocalisation of endocytosed mitochondria with endosomes and lysosomes at each time established that a majority of these exogenous organelles were associated with these vesicles at the earlier times (0.5 to 2 h); however, there was no obvious pattern to endolysosomal trafficking other than a decline in colocalisation at 4 h (Fig. 3b). The number of mitochondria escaping from these compartments showed the opposite trend with the largest number of organelles emerging from late endosomes and lysosomes at 4 h (Fig. 3c). We extended these analyses to include colocalisation between endogenous and exogenous mitochondria (Fig. 3d). These experiments demonstrated a large percentage of internalised GFP-labelled mitochondria were closely associated with the cardiomyocyte mitochondrial network from 0.5 to 4 h. The functional consequence of the uptake and incorporation of extracellular mitochondria in cardiomyocytes was a sustained and significant increase in ATP production, similar to our earlier findings^9^. Together, these results signify that endocytosis and intracellular transportation of extracellular mitochondria in cardiomyocytes is an ongoing and efficient biological process.

### Fusion of exogenous mitochondria with the endogenous mitochondrial network

**Figure 4:**
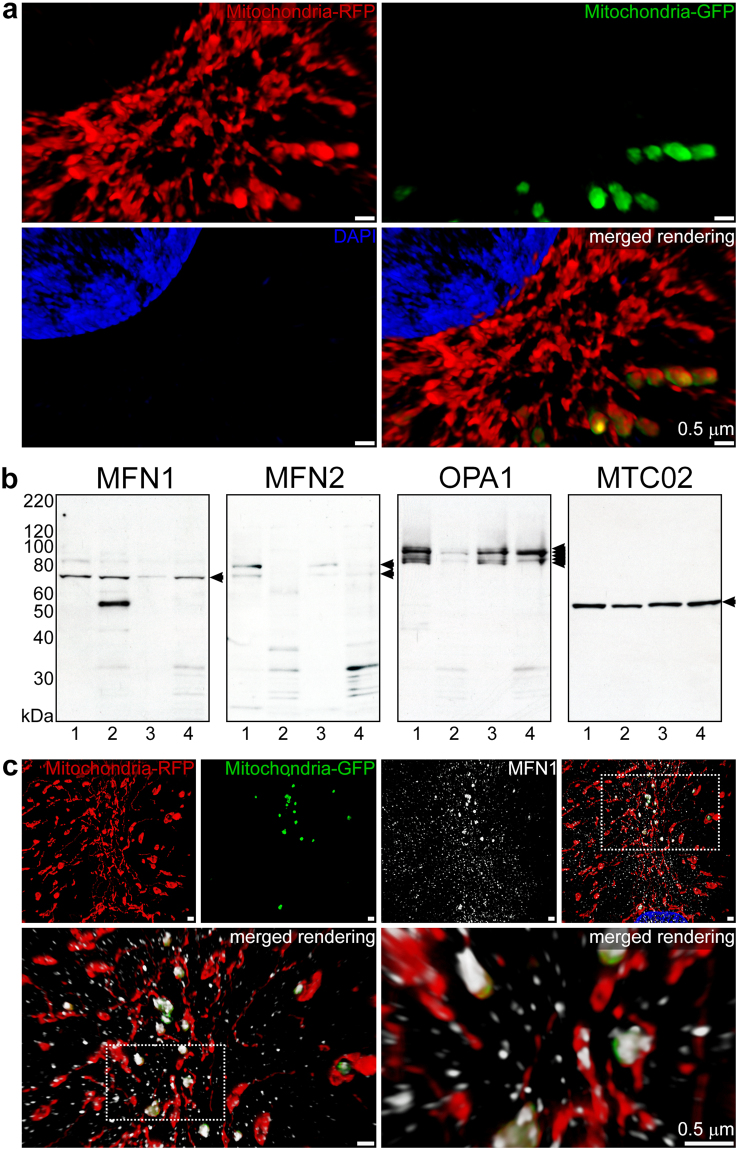
Fusion of exogenous mitochondria with the cardiomyocyte mitochondrial network. (**a**) iPS-CMs with RFP-labelled mitochondria (red) treated for 4 h with isolated GFP-labelled HCF mitochondria (green). After fixation, the nuclei were stained with DAPI (blue) and slides were imaged using 3-D SR-SIM. Individual red, green, and blue channels are shown along with the combined color rendering. Fusion of endocytosed mitochondria with the endogenous mitochondrial network is readily apparent. Mitochondrial fusion was apparent at all times examined (0.5, 1, 2, and 4 h) and four separate experiments were performed. Scale bars, 0.5 µm. (**b)** Immunoblot analysis of whole cell and isolated mitochondrial lysates (25 µg per lane) from iPS-CMs and HCFs using antibodies directed against MFN1, MFN2, OPA1, and mitochondria (MTC02). Lanes 1 to 4 contained the following lysates: iPS-CM cell proteins, HCF cell proteins, iPS-CM mitochondrial proteins, and HCF mitochondrial proteins. Arrows indicate the specifically detected protein(s) for each antibody and immunoblot experiments were repeated six times. (**c)** 3-D SR-SIM of human cardiomyocytes containing RFP-labelled mitochondria (red) incubated with isolated GFP-labelled exogenous mitochondria (green) for 30 min. Coverslips were fixed and stained with a mitofusin-1 (MFN1) antibody (white). The top panels show the three separate color channels and the combined image (left to right). The boxed regions have been magnified in successive images (top right to lower panels, left to right) to show the reactivity of the MFN1 antibody with fusing mitochondria and the presence of this antibody throughout the cytosol. Parallel experiments were performed at 1, 2, and 4 h and the presented images are representative of four separate experiments. Scale bars, 0.5 µm.

The fusion of endocytosed mitochondria with the endogenous mitochondria of cardiomyocytes (Fig. 4a and Movie S3) and cardiac fibroblasts (Extended Data Fig. 3) was assessed by 3-D SR-SIM. In combination with depth coding analyses (Extended Data Fig. 4a and Extended Data Fig. 3a), these experiments demonstrated that exogenous mitochondria fused with the endogenous mitochondrial network after escaping from endolysosomal compartments at 0.5, 1, 2, and 4 h. The presence of mitochondrial fusion-associated proteins was assessed by studying the expression of mitofusin-1 (MFN1), mitofusin-2 (MFN2), and optic atrophy 1 (OPA1) proteins in iPS-CMs and HCFs (Fig. 4b). Whole cell and isolated mitochondrial lysates were analysed for fusion proteins by immunoblot analyses. The expression and phosphorylation of the mitochondrial fission-associated dynamin-related protein 1 (DRP1) and the presence of the mitophagy-associated protein Parkin (Extended Data Fig. 4b) were determined for comparison. The anti-mitochondria antibodies MTC02 and 113-1 were used to establish equivalent loading of lysates in polyacrylamide gels^8^. We subsequently examined the spatial distribution of mitofusin proteins in cardiomyocytes using 3-D SR-SIM (Fig. 4c, Extended Data Fig. 4c, and Movie S4).

Whole cell lysates from iPS-CMs and HCFs contained equivalent amounts of MFN1 protein (Fig. 4b, lanes 1 and 2); however, only cardiomyocytes had detectable quantities of MFN2 (Fig. 4b, lane 1). In contrast, examination of lysates from isolated mitochondria revealed cardiomyocytes had relatively less MFN1 associated with these organelles (Fig. 4b, lane 3). HCFs had nearly equal levels of this protein in whole cell and mitochondrial lysates (Fig. 4b, lanes 2 and 4). It is worth noting that the MFN1 antibody strongly reacted with an approximately 55 kDa protein in HCF whole cell lysates. This could indicate the presence of a protein related to MFN1 in HCFs that was not associated with mitochondria. Isolated mitochondria from both cell types contained low levels of MFN2 (Fig. 4b, lanes 3 and 4). Analysis of OPA1 expression showed cardiomyocytes possessed relatively large quantities of long and short isoforms of this protein, whereas HCFs produced fewer variants in reduced quantities (Fig. 4b, lanes 1 and 3 versus 2 and 4). A comparison of whole cell lysates with mitochondrial lysates confirmed OPA1 was largely associated with mitochondria (Fig. 4b, lanes 1 and 2 versus lanes 3 and 4). Identical immunoblots confirmed equal reactivity of each lysate with MTC02 (Fig. 4b) and 113–1 (Extended Data Fig. 4b). DRP1 and a phosphorylated variant of DRP1 (S616) were expressed in both iPS-CMs and HCFs (Extended Data Fig. 4b, lanes 1 and 2). Evaluation of mitochondrial lysates revealed a small amount of DRP1 protein in iPS-CMs (Extended Data Fig. 4b, lane 3); but, S616 phosphorylated DRP1 was not observed (Extended Data Fig. 4b, lane 3). Whole cell and mitochondrial lysates from fibroblasts contained nearly equal levels of DRP1 (Extended Data Fig. 4b, lane 2 and 4) and DRP1 S616 (Extended Data Fig. 4b, lanes 2 and 4). Additional immunoblot analysis of DRP1 S637 phosphorylation using the same protein lysates failed to detect this post-translational modification (not shown); however, Parkin was present in whole cell lysates from HCFs (Extended Data Fig. 4b, lane 2).

Imaging of the spatial distribution of MFN1 and MFN2 using 3-D SR-SIM demonstrated that these proteins localised to fusing exogenous and endogenous mitochondria in addition to a multitude of other intracellular sites (Fig. 4c and Extended Data Fig. 4c). Even though MFN1 and MFN2 were observed to be concentrated at the sites of fusion (Fig. 4c, Movie S4, and Extended Data Fig. 4c), both mitofusin proteins were widely dispersed throughout the cytosol and nuclei of cardiomyocytes.

Together, our results prove that extracellular mitochondria are rapidly and efficiently endocytosed in human iPS-CMs and primary HCFs and the majority of these exogenous organelles fuse with the endogenous mitochondrial network (Extended Data Fig. 5). Cardiomyocyte and fibroblast mitochondria contain fusion-associated proteins (*e.g*. MFN1, MFN2, and OPA1), which probably facilitates this process. At the same time, some exogenous mitochondria do not appear to escape from endolysosomal compartments and presumably undergo hydrolysis and degradation through autophagy—an assumption supported by the appearance of fragmented mitochondria in some lysosomes (Extended Data Fig. 2d).

## Discussion

The ability to distinguish the precise intracellular location of internalised exogenous human mitochondria using 3-D super-resolution microscopy and transmission electron microscopy in cardiac cells has allowed us to understand the intracellular trafficking and fate of these organelles through time. Electron microscopy revealed that extracellular mitochondria begin to be endocytosed by human iPS-derived cardiomyocytes (iPS-CMs) and primary human cardiac fibroblasts (HCFs) within minutes. These exogenous organelles successively progress through the endolysosomal system from early endosomes to late endosomes to lysosomes. While some of these mitochondria appear to be destined for degradation, our results provide compelling evidence that the majority of exogenous mitochondria escape from endosomal and lysosomal compartments and effectively fuse with endogenous cardiac cell mitochondria. Our findings in iPS-CMs and HCFs support the idea that endocytosis and integration of exogenous mitochondria is an evolutionarily-conserved biological process that is distinct from recently described cell-to-cell transfer of mitochondria through tunneling nanotubes or exosomes^16,26–28^. In essence, our observations of the uptake of isolated mitochondria in human heart cells corroborates the endosymbiotic theory advanced and substantiated by Lynn Margulis (née Sagan) half a century ago^29^.

Our earlier experiments, and those of others, established that endocytosis of extracellular mitochondria was dependent on actin polymerisation^9,11,14^ and that internalisation of these organelles resulted in long-term replenishment of mtDNA in HeLa cell-derived ρ^0^ cells^9,28,30^, which are devoid of mtDNA^31^. Restoration of the ρ^0^ mitochondrial genome through the uptake of mitochondria isolated from HeLa cells led to enhancement of intracellular ATP levels and oxygen consumption rates. Because increases in ρ^0^ cellular respiration persisted for more than 50 population doublings, it was evident that endocytosed HeLa cell mitochondria containing functional mtDNA were lastingly integrated within these cells^9^. In spite of this discovery, we did not resolve how internalised HeLa cell mitochondria were transported within ρ^0^ cells or determine whether they fused with endogenous mitochondria, persisted as distinct organelles, or merely contributed their DNA to ρ^0^ mitochondria. The same can be said for our previous experiments, which established internalisation of extracellular mitochondria in cardiac cells^7,8^.

In this study, we defined the intracellular transit of extracellular mitochondria from the plasma membrane through to their incorporation with the endogenous mitochondrial network in two types of human heart cells—dividing HCFs and non-dividing iPS-CMs. Purified iPS-CMs represent a suitable experimental model as these spontaneously-beating cells do not divide and retain the biochemical and electrophysiological responses typical of human cardiac myocytes. We anticipated that these terminally differentiated cells would more accurately predict *in vivo* responses compared to other culture models like immortalised cells or cardiac progenitors, which exhibit a less definitive differentiation potential^32^. Because iPS-CMs were reprogrammed using non-integrating episomal vectors and cultured without feeder cells, they had a consistent phenotype with no pathogenic contaminants. At the same time, our cardiomyocyte cultures displayed a uniform ‘late phase’ cardiomyocyte phenotype with a well-developed mitochondrial network and organized contractile apparatus^33^. For comparison, we examined primary HCFs, which were not derived from iPS cells^7^. We found that both cell types could efficiently internalise extracellular mitochondria and incorporate these organelles into the endogenous mitochondrial network.

Our findings suggest that several proteins are involved in fusion of exogenous mitochondria with endogenous mitochondria in iPS-CMs and HCFs. Mitofusin proteins are involved in tethering and fusing the mitochondrial outer membranes and OPA1 functions, in part, to enable inner mitochondrial membrane fusion^34–36^. Our results show that MFN1, MFN2, and OPA1 are expressed in both cardiomyocytes and fibroblasts and that a sizeable proportion of these proteins are associated with their respective mitochondria. Though mitofusin proteins concentrate at sites where exogenous and endogenous mitochondria are undergoing fusion, the presence of MFN1 and MFN2 beyond the boundaries of the mitochondrial network indicate these proteins may be involved in performing other cellular roles. In contrast, our immunoblot results reveal that OPA1 remains predominantly confined to the mitochondrial fraction and at least five OPA1 isoforms exist in cardiomyocytes. The long forms of OPA1 are membrane-anchored proteins involved in fusion of the mitochondrial inner membrane, whereas the short forms are soluble proteins associated with mitochondrial fission and other functions^37^.

To study fission-associated proteins in these cells, we examined the expression and post-translational modification of DRP1 by immunoblot analysis. DRP1 is a GTPase that oligomerises into structures that constrict and cleave mitochondria at specific sites on the outer membrane^35^. Serine phosphorylation of this protein regulates its activity^38^. For example, phosphorylation of DRP1 at S616 promotes mitochondrial fission during mitosis, whereas phosphorylation of S637 inhibits GTPase activity and fission^39,40^. Dephosphorylation of the latter amino acid promotes mitochondrial fission. The presence of fusion and fission proteins in iPS-CMs and HCFs is compatible with normal mitochondrial dynamics and our results confirm these cardiac cells contain the machinery necessary for fusion of exogenous mitochondria with the endogenous mitochondrial network. Given the metabolic and mitotic differences in these cells, it is not surprising to see variable expression and modification of these proteins. Interestingly, only HCFs expressed detectable levels of the mitophagy-related protein Parkin. Parkin is an E3 ubiquitin ligase that functions in a signaling pathway that identifies and eliminates dysfunctional mitochondria^41,42^. The absence of this protein in isolated mitochondrial lysates suggests there was little mitophagy occurring in either iPS-CMs or HCFs.

A complete understanding of the mechanisms that bring about endocytosis of extracellular mitochondria and fusion of those organelles with the endogenous mitochondrial network may provide a foundation for innovative new treatments to a number of human diseases and disorders^43^. Our earlier work has supported the use of mitochondrial transplantation to decrease infarction, increase ATP content, and improve functional performance of the ischemic heart^6–9,17^. We also demonstrated that delivery of exogenous mitochondria to the heart in animal models does not provoke an immune response nor produce arrhythmias^7,21^. Importantly, the clinical relevance and safety of autologous mitochondrial transplantation to the ischemic heart has recently been validated in five pediatric patients^44^. Aside from providing benefits to ischemic tissues, transplantation of exogenous mitochondria may prove to be useful for treating mitochondrial myopathies and a number of other diseases with underlying mitochondrial dysfunction^13,15–18,20,45–48^. Further study of the molecular mechanisms of exogenous mitochondrial endocytosis and fusion with endogenous mitochondria are warranted and these inquiries may guide development of innovative treatments directed at augmenting impaired mitochondria in a wide range of tissues and cells.

## Methods

### Reagents

The following antibodies were purchase from Abcam: anti-α-actinin (ab68167), anti-vimentin (ab92547), anti-TOMM20 (ab56783), anti-mitochondria [MTC02] (ab3298), anti-mitochondria [113-1] (ab92824), anti-Mitofusin 1 [MFN1] (ab57602 and ab104274), anti-Mitofusin 2 [MFN2] (ab56889 and ab101055), anti-OPA1 (ab90857), anti-Parkin (ab15954), anti-DRP1 [EPR19275] (ab184248), anti-DRP1 [phospho S637] (ab193216) and anti-Rab7 (ab50533). The anti-phosphoDRP1 Ser616 [D9A1] antibody (CST-4494S) was purchased from Cell Signaling. The mouse IgG isotype control (10400 C) and rabbit IgG isotype control (10500 C) antibodies were purchased from ThermoFisher Scientific. The following CellLight reagents were purchased from ThermoFisher Scientific: Early endosomes (C10586 and C10587), Endoplasmic Reticulum (C10590 and C10591), Golgi Complex (C10592 and C10593), Late endosomes (C10588 and C10589), Lysosomes (C10596 and C10597), Mitochondria (C10600 and C10601) and Null Virus Negative Control (C10615). MitoTracker Red CMXRos (M7512) was purchased from ThermoFisher Scientific and used according to the manufacturer’s directions at 100 nM.

### Cells

iCell-Cardiomyocytes^2^ (iPS-CMs) (CMC-100-012-001) were purchased from Cellular Dynamics and Human Cardiac Fibroblasts (HCFs) (6300) were purchased from Sciencell Research Laboratories. Cells were cultured according to the manufacturer’s directions. Baculovirus infections were performed on dividing HCFs by adding 400 μL of CellLight reagent to a 100-mm confluent plate of cells for 16 h. Spontaneously contracting syncytial monolayers of iPS-CMs (≥96 hours after plating) were infected for 16 h by using 10% CellLight reagent in iCell Cardiomyocyte Maintenance Medium (CMM-100-120-001) (Cellular Dynamics) containing 0.2 μM BacMam Enhancer (ThermoFisher Scientific).

### Mitochondria

HCF media was replaced with 4 mL of 4 °C homogenisation buffer (300 mM sucrose, 10 mM HEPES-KOH, 1 mM EGTA-KOH, pH 7.4) containing 2 mg Subtilisin A protease from *Bacillus licheniformis* (Sigma-Aldrich) and incubated at room temperature for 5 min. Digested cells were transferred to a 15-mL tube on ice and digested for an additional 15 min before filtration through a 10 μm mesh filter (PluriSelect) saturated with cold homogenisation buffer. Mitochondria were collected by centrifugation at 9,500 RCF at 4 °C for 5 minutes and washed 3 times in cold homogenisation buffer before resuspension in culture media. Mitochondrial number was determined by using a Multisizer 4e Coulter Counter (Beckman-Coulter) and corroborated by hemocytometry. Isolated mitochondria were incubated with HCFs or iPS-CMs for 0 to 4 h.

### Labelling

Some isolated mitochondria (1 × 10^8^) were conjugated to 10 nm NHS-activated gold nanoparticles (CytoDiagnostics) according to the manufacturer’s directions. Labeled mitochondria were washed 5 times with homogenisation buffer containing 1 mg/mL fraction V bovine serum albumin (BSA) to remove unreacted nanoparticles. Gold-labeled mitochondria were incubated with iPS-CMs for 0, 2.5, 5 and 10 minutes prior to fixation and preparation for transmission electron microscopy.

### Immunoblots

Whole cell proteins were isolated by rinsing cells with ice-cold PBS (pH 7.4) and lifting them from the plates with a rubber policeman. Mitochondrial proteins were isolated by rinsing the mitochondrial pellet with ice-cold PBS (pH 7.4) five times. Pelleted cells or isolated mitochondria were then resuspended in a small volume of freshly-prepared ice-cold lysis solution (150 mM NaCl, 20 mM Tris-HCl (pH 7.6), 1 mM EDTA, 0.5% sodium deoxycholate, 70 mM NaF, 1% Nonidet P-40, Complete protease inhibitor cocktail (Sigma-Aldrich), 200 μM sodium orthovanadate, and 2 μM phenylmethylsulfonyl fluoride)^49^. After a 10-min incubation on ice with intermittent, brief agitation, debris was pelleted in a microcentrifuge, and the supernatants were stored at −80 °C. Protein concentrations were determined using the bicinchoninic acid (BCA) protein determination kit (Pierce). SDS-PAGE and transfer to nitrocellulose was performed using the XCell SureLock Mini-Cell System and XCell II Blot Module (ThermoFisher), respectively. Identical 4–20% Tris-Glycine gels (ThermoFisher) were stained with Coomassie brilliant blue R250 to confirm equal protein loading. The ColorBurst Electrophoresis Marker (Sigma-Aldrich) and MagicMark XP Western Protein Standard (ThermoFisher) were used according to the manufacturer’s directions. Nitrocellulose membranes were rinsed in Tris-buffered saline (pH 7.4) containing 0.1% Tween 20 (TBS-T) and blocked in 5% non-fat milk in TBS-T for 1 h at 22 °C on a rocking platform. Membranes were incubated overnight at 4 °C on an orbital shaker with primary antibodies diluted according to the manufacturer’s directions in TBS-T containing 1% non-fat milk (Sigma). Primary antibodies were detected with the Amersham ECL Western Blotting Analysis System (GE Healthcare) using HRP-conjugated secondary antibodies (GE-Healthcare).

### Staining

Cells cultured on No 1.5 H (170 μm ± 5 μm) coverslips (Marienfeld-Superior) were fixed in 4% freshly-prepared formaldehyde in phosphate buffered saline (PBS). Cells were permeabilised in 0.1% Triton X-100 in PBS for 3 minutes and incubated with primary antibodies diluted 1:1000 in 10% fetal bovine serum (FBS) in PBS for 1 h. Primary antibodies were detected with species-appropriate Alexa Fluor 488-, 568-, or 633-conjugated secondary antibodies (ThermoFisher) diluted in PBS and cells were simultaneously stained using 4′,6-diamidino-2-phenylindole (DAPI) (ThermoFisher). Coverslips were mounted to slides using ProLong Diamond Mountant (ThermoFisher).

### Microscopy

Cells were visualised on a ELYRA PS.1 (Zeiss) attached to a LSM 710 inverted microscope (Zeiss) with a 100x oil objective (Zeiss) and a DAPI/GFP/mRFP/Alexa 633 fluorescence filter set. Optical sections (84 nm) were collected through entire cell volumes using 5 grid patterns for structured illumination microscopy. Image stacks were processed using ZEN Black software (Zeiss) and displayed as transparent three-dimensional (3-D) volumetric renderings. Widefield fluorescence microscopy was performed using a FSX100 (Olympus). For transmission electron microscopy, iPS-CMs cultured on Thermanox coverslips (ThermoFisher) were fixed in 2% formaldehyde, 2.5% grade I glutaraldehyde, and 0.03% picric acid in 0.1 M cacodylate buffer, pH 7.4 at 4 °C prior to incubation in 1% osmium tetroxide and 1.5% potassium ferrocyanide dissolved in cacodylate buffer. After contrast staining with 1% uranyl acetate in cacodylate buffer, cells were dehydrated and infiltrated with a 1:1 mixture of Epon-Araldite (Electron Microscopy Sciences) and propylene oxide. Embedding resins were polymerised for 24 to 48 h at 60 °C and 60 nm-thick sections on were placed on copper grids and imaged on a Jeol 1200EX (80 kV).

### Biochemistry

ATP content was determined in the presence or absence of 1 μM adenosine diphosphate (ADP) using the ATPlite luminescence assay (PerkinElmer).

### Statistics

Control and experimental ATP data was expressed as concentration ± standard error of the mean (SEM). Statistical analysis was performed by Student’s t-test and a p < 0.05 was considered significant. The number of internalised GFP-labeled mitochondria associated with RFP-labeled early endosomes, late endosomes, lysosomes, or mitochondria was enumerated at 0.5, 1, 2, and 4 h by fluorescence microscopy. Randomly-selected, high-powered (400x) fields were acquired at each time point and in each of the 4 cell compartments. Between 1,852 to 12,007 mitochondria were counted per compartment by assessing anywhere from 100 to 473 individual cells at any given time (0.5, 1, 2, and 4 h). The raw data for these analyses is presented in Extended Data Fig. 6. For early endosomes, late endosomes and lysosomes, GFP-labeled mitochondria were quantified as fully encapsulated, partially encapsulated (escaping) or not associated with each compartment. GFP-mitochondria were determined to be either fusing with the RFP-labeled endogenous mitochondria or not associated with these organelles. Analysis of the cell count data was subsequently performed using a generalised linear modeling approach with the gamma distribution and a log link function to evaluate the escaping mitochondria as a percentage of total cells between the 4 time points (0.5, 1, 2, and 4 h) within each compartment (early endosome, late endosome, and lysosome)^50^. Fit of the model was assessed by the Akaike information criterion. Summary data are expressed in terms of the mean percentage with the standard error and two-tailed Bonferroni adjusted. p < 0.05 are considered statistically significant based on the Wald test. Statistical analysis was conducted using SPSS Statistics version 23.0 (IBM).

### Data Availability

The datasets generated during and/or analysed during the current study are available from the corresponding author on reasonable request.

## Electronic supplementary material

Supplementary Information

Movie S1

Movie S2

Movie S3

Movie S4

## References

[1] Ozcan C, Holmuhamedov EL, Jahangir A, Terzic A (2001). Diazoxide protects mitochondria from anoxic injury: Implications for myopreservation. J. Thorac. Cardiovasc. Surg..

[2] McCully JD (2002). Diazoxide amelioration of myocardial injury and mitochondrial damage during cardiac surgery. Ann. Thorac. Surg..

[3] Lesnefsky EJ (2004). Ischemia, rather than reperfusion, inhibits respiration through cytochrome oxidase in the isolated, perfused rabbit heart: Role of cardiolipin. Am. J. Physiol. Heart Circ. Physiol..

[4] Rousou AJ, Ericsson M, Federman M, Levitsky S, McCully JD (2004). Opening of mitochondrial KATP channels enhances cardioprotection through the modulation of mitochondrial matrix volume, calcium accumulation, and respiration. Am. J. Physiol. Heart Circ. Physiol..

[5] Kornfeld OS (2015). Mitochondrial reactive oxygen species at the heart of the matter: New therapeutic approaches for cardiovascular diseases. Circ. Res..

[6] McCully JD (2009). Injection of isolated mitochondria during early reperfusion for cardioprotection. Am. J. Physiol. Heart Circ. Physiol..

[7] Masuzawa A (2013). Transplantation of autologously derived mitochondria protects the heart from ischemia-reperfusion injury. Am. J. Physiol. Heart Circ. Physiol..

[8] Cowan DB (2016). Intracoronary delivery of mitochondria to the ischemic heart for cardioprotection. PLoS ONE.

[9] Pacak CA (2015). Actin-dependent mitochondrial internalization in cardiomyocytes: Evidence for rescue of mitochondrial function. Biol. Open.

[10] Clark MA, Shay JW (1982). Mitochondrial transformation of mammalian cells. Nature.

[11] Kitani T, Kami D, Matoba S, Gojo S (2014). Internalization of isolated functional mitochondria: Involvement of macropinocytosis. J. Cell. Mol. Med..

[12] Kitani T (2014). Direct human mitochondrial transfer: A novel concept based on the endosymbiotic theory. Transplant. Proc..

[13] Caicedo A (2015). MitoCeption as a new tool to assess the effects of mesenchymal stem/stromal cell mitochondria on cancer cell metabolism and function. Sci. Rep..

[14] Kesner EE, Saada-Reich A, Lorberboum-Galski H (2016). Characteristics of mitochondrial transformation into human cells. Sci. Rep..

[15] Hayakawa K (2016). Transfer of mitochondria from astrocytes to neurons after stroke. Nature.

[16] Islam MN (2012). Mitochondrial transfer from bone-marrow-derived stromal cells to pulmonary alveoli protects against acute lung injury. Nat. Med..

[17] McCully JD, Levitsky S, del Nido PJ, Cowan DB (2016). Mitochondrial transplantation for therapeutic use. Clin. Transl. Med..

[18] Exner N, Lutz AK, Haass C, Winklhofer KF (2012). Mitochondrial dysfunction in Parkinson’s disease: Molecular mechanisms and pathophysiological consequences. EMBO J..

[19] Agrawal A, Mabalirajan U (2016). Rejuvenating cellular respiration for optimizing respiratory function: Targeting mitochondria. Am. J. Physiol. Lung Cell. Mol. Physiol..

[20] Liu CS (2014). Delivering healthy mitochondria for the therapy of mitochondrial diseases and beyond. Int. J. Biochem. Cell. Biol..

[21] Kaza AK (2017). Myocardial rescue with autologous mitochondrial transplantation in a porcine model of ischemia/reperfusion. J. Thorac. Cardiovasc. Surg..

[22] Kumari S, Mg S, Mayor S (2010). Endocytosis unplugged: Multiple ways to enter the cell. Cell Res..

[23] Guerra, F. & Bucci, C. Multiple roles of the small GTPase Rab7. *Cells* **5** (2016). 10.3390/cells5030034 PMC5040976 27548222

[24] Bucci C, Thomsen P, Nicoziani P, McCarthy J, van Deurs B (2000). Rab7: A key to lysosome biogenesis. Mol. Biol. Cell.

[25] Humphries WHT, Szymanski CJ, Payne CK (2011). Endo-lysosomal vesicles positive for Rab7 and LAMP1 are terminal vesicles for the transport of dextran. PLoS ONE.

[26] Plotnikov EY, Khryapenkova TG, Galkina SI, Sukhikh GT, Zorov DB (2010). Cytoplasm and organelle transfer between mesenchymal multipotent stromal cells and renal tubular cells in co-culture. Exp. Cell Res..

[27] Torralba D, Baixauli F, Sanchez-Madrid F (2016). Mitochondria know no boundaries: Mechanisms and functions of intercellular mitochondrial transfer. Front. Cell Dev. Biol..

[28] Lin HY (2015). Mitochondrial transfer from Wharton’s jelly-derived mesenchymal stem cells to mitochondria-defective cells recaptures impaired mitochondrial function. Mitochondrion.

[29] Sagan L (1967). On the origin of mitosing cells. J. Theor. Biol..

[30] Wu TH (2016). Mitochondrial transfer by photothermal nanoblade restores metabolite profile in mammalian cells. Cell Metab..

[31] Kukat A (2008). Generation of rho0 cells utilizing a mitochondrially targeted restriction endonuclease and comparative analyses. Nucleic Acids Res..

[32] Citro L (2014). Comparison of human induced pluripotent stem-cell derived cardiomyocytes with human mesenchymal stem cells following acute myocardial infarction. PLoS ONE.

[33] Robertson C, Tran DD, George SC (2013). Concise review: Maturation phases of human pluripotent stem cell-derived cardiomyocytes. Stem Cells.

[34] Franco A (2016). Correcting mitochondrial fusion by manipulating mitofusin conformations. Nature.

[35] Pernas L, Scorrano L (2016). Mito-Morphosis: Mitochondrial fusion, fission, and cristae remodeling as key mediators of cellular function. Annu. Rev. Physiol..

[36] Mishra P, Chan DC (2016). Metabolic regulation of mitochondrial dynamics. J. Cell Biol..

[37] MacVicar T, Langer T (2016). OPA1 processing in cell death and disease - the long and short of it. J. Cell Sci..

[38] Elgass K, Pakay J, Ryan MT, Palmer CS (2013). Recent advances into the understanding of mitochondrial fission. Biochim. Biophys. Acta.

[39] Taguchi N, Ishihara N, Jofuku A, Oka T, Mihara K (2007). Mitotic phosphorylation of dynamin-related GTPase Drp1 participates in mitochondrial fission. J. Biol. Chem..

[40] Liesa M, Palacin M, Zorzano A (2009). Mitochondrial dynamics in mammalian health and disease. Physiol. Rev..

[41] Burman, J. L. *et al*. Mitochondrial fission facilitates the selective mitophagy of protein aggregates. *J. Cell Biol*. 10.1083/jcb.201612106 (2017). 10.1083/jcb.201612106 PMC5626535 28893839

[42] Pickrell AM, Youle RJ (2015). The roles of PINK1, parkin, and mitochondrial fidelity in Parkinson’s disease. Neuron.

[43] McCully JD, Cowan DB, Emani SM, del Nido PJ (2017). Mitochondrial transplantation: From animal models to clinical use in humans. Mitochondrion.

[44] Emani SM, Piekarski BL, Harrild D, del Nido PJ, McCully JD (2017). Autologous mitochondrial transplantation for dysfunction after ischemia-reperfusion injury. J. Thorac. Cardiovasc. Surg..

[45] Kumar SR (2017). Mitochondrial transplantation: Another miracle of molecular medicine?. J. Thorac. Cardiovasc. Surg..

[46] Robicsek, O. *et al*. Isolated mitochondria transfer improves neuronal differentiation of schizophrenia-derived induced pluripotent stem cells and rescues deficits in a rat model of the disorder. *Schizophr. Bull*. 10.1093/schbul/sbx077 (2017). 10.1093/schbul/sbx077 PMC5814822 28586483

[47] Singh, B., Modica-Napolitano, J. S. & Singh, K. K. Defining the momiome: Promiscuous information transfer by mobile mitochondria and the mitochondrial genome. *Semin. Cancer Biol*. 10.1016/j.semcancer.2017.05.004 (2017). 10.1016/j.semcancer.2017.05.004 PMC5681893 28502611

[48] Chang JC (2013). Treatment of human cells derived from MERRF syndrome by peptide-mediated mitochondrial delivery. Cytotherapy.

[49] Cowan DB, Poutias DN, del Nido PJ, McGowan FX (2000). CD14-independent activation of cardiomyocyte signal transduction by bacterial endotoxin. Am. J. Physiol. Heart Circ. Physiol..

[50] McCullagh, P. & Nelder, J. A. *Generalized linear models*. 2nd edn, 285–322 (Chapman and Hall 1989).

