# Supplementary material for: Transit and integration of extracellular mitochondria in human heart cells
Source: Sci Rep. 2017 Dec 12;7:17450. doi: 10.1038/s41598-017-17813-0 (PMC5727261; doi:10.1038/s41598-017-17813-0)
Supplement: Supplementary file 1 — Supplementary Information [file 41598_2017_17813_MOESM1_ESM.pdf]

Transit and integration of extracellular mitochondria in human heart cells

Douglas B. Cowan[1,2,3], Rouan Yao[1], Jerusha K. Thedsanamoorthy[1], David Zurakowski[1,2,4], Pedro J. del Nido[4,5] & James D. McCully[4,5]

[1]Department of Anesthesiology, Perioperative and Pain Medicine, Boston Children's Hospital, Boston, Massachusetts 02115, USA.

[2]Department of Anæsthesia, Harvard Medical School, Boston, Massachusetts 02115, USA.

[3]Harvard Stem Cell Institute, Cambridge, Massachusetts 02138, USA.

[4]Department of Cardiac Surgery, Boston Children's Hospital, Boston, Massachusetts 02115, USA.

[5]Department of Surgery, Harvard Medical School, Boston, Massachusetts 02115, USA.

Corresponding author - Douglas B. Cowan, Boston Children's Hospital, 300 Longwood Avenue, Enders 312.1, Boston, MA 02115-5724 USA. Telephone – (617) 919-2655, E-mail – douglas.cowan@childrens.harvard.edu

Supplementary Information

METHODS

Movies. Three-dimensional (3-D) SR-SIM image stacks were acquired on the ELYRA PS.1 microscope (Zeiss) and processed using ZEN Black software (Zeiss). Image stacks were imported into Volocity 6.3 software (PerkinElmer) to generate animated volumetric renderings.

FIGURE LEGENDS

Extended Data Figure 1 | Widefield fluorescence microscopy of human cardiomyocytes and cardiac fibroblasts. a, iPS-CMs (left) and HCFs (right) stained with ACTN or vimentin antibodies (red), respectively. Mitochondria in these cells (green) were stained with MTC02 (left) or TOMM20 (right) and the nuclei were stained with DAPI (blue). Scale bars, 50 µm. **b,** Cardiomyocytes (top panels) and fibroblasts (bottom panels) infected with various CellLight BacMam 2.0 reagents. Baculovirus infection rates after 16 h were similar for each cell type (84.45% ± 1.45 for iPS-CMs and 84.43% ± 1.80 for HCFs, mean percentage of cells expressing fluorescent proteins ± SEM). Expression of RFP was apparent in the appropriate cellular organelles. Images represent six separate infections with each baculovirus reagent. Scale bars, 10 µm.

Extended Data Figure 2 | Internalised exogenous mitochondrial position within cardiomyocytes. a, An exogenous mitochondria (green) associated with the endogenous mitochondrial network (red) after 1 h in iPS-CMs that were subsequently stained for the contractile apparatus with ACTN (white) and DNA with DAPI (blue). The combined image (left) and individual colors (right) are shown and images are representative of four separate experiments. Scale bar, 10 µm. **b,** Early endosomes (red), internalised mitochondria (green), and EEA1 staining (white) of iPS-CMs at 30 min. These images show exogenous mitochondria

encapsulated within early endosomes, escaping from these vesicles, or not associated with this compartment. Images are representative of four separate experiments. Scale bars, 0.5 µm. **c,** Late endosomes (red), internalised mitochondria (green), and Rab7 staining (white) of iPS-CMs at 2 h. These panels show exogenous mitochondria encapsulated and escaping from late endosomes. Images are representative of four separate experiments. Scale bars, 0.5 µm. **d,** Lysosomes (red), internalised mitochondria (green), and Rab7 staining (white) of iPS-CMs at 2 h. The encapsulated GFP-labelled mitochondria appear to be fragmented, indicative of hydrolytic enzyme activity. Images are representative of four separate experiments. Scale bars, 0.5 µm.

Extended Data Figure 3 | Exogenous mitochondrial fusion with endogenous mitochondria in human cardiac fibroblasts. a, RFP-labelled HCF mitochondria were isolated and incubated with HCFs containing GFP-labelled mitochondria for 2 h. The transparent rendering was depicted with a black (left panel) and grey background (middle panel) to accentuate the association of exogenous mitochondria with the endogenous mitochondrial network (arrows). Depth coding analysis using Zen Black software (Zeiss) showed that RFP and GFP mitochondria occupied the same spatial position in the 1.6 µm thick cell. The color scale indicates distance (µm) from the bottom to the top of the cell and images are representative of four separate experiments. Scale bars, 10 µm. **b,** a 3-D volumetric rendering (left) and rotation (right) of a fibroblast containing RFP-labelled mitochondria that had been exposed to isolated GFP-labelled HCF extracellular mitochondria for 4 h. Fusion of exogenous and endogenous mitochondria was apparent (arrows). Images are representative of four separate experiments and similar results were obtained at 0.5, 1, and 2 h. Scale bars, 10 µm.

Extended Data Figure 4 | Depth-coding analysis of fusion in iPS-CMs and involvement of mitofusin-2. a, Depth coding analysis of the images presented in Fig 4a using Zen Black

software (Zeiss). The color scale extends for 2.5 μm and the fusing mitochondria occupied identical spatial positions as the endogenous mitochondrial network. **b,** immunoblot analysis of whole cell and isolated mitochondrial lysates (25 μg per lane) from iPS-CMs and HCFs using antibodies directed against DRP1, DRP1 phosphorylated at serine residue 616 (DRP1 S616), Parkin, and mitochondria (113-1). Lanes 1 to 4 contained the following lysates: iPS-CM cell proteins, HCF cell proteins, iPS-CM mitochondrial proteins, and HCF mitochondrial proteins. **c,** 3-D SR-SIM of human cardiomyocytes containing RFP-labelled mitochondria (red) incubated with isolated GFP-labelled exogenous mitochondria (green) for 2 h. Coverslips were fixed and stained with a mitofusin-2 (MFN2) antibody (white). The top and bottom panels show separate color channels and the combined images at two different magnifications (left to right). Although the antibody reacted with fusing mitochondria, there was considerable staining of the nucleus and cytosol that was not associated with mitochondria. Images are representative of four separate experiments. Scale bars, 10 μm (top) and 0.5 μm (bottom).

Extended Data Figure 5 | Schematic representation of the fate of endocytosed mitochondria. Extracellular mitochondria are internalised in human cardiomyocytes and cardiac fibroblasts through actin-dependent endocytosis. The majority of exogenous mitochondria escape from endosomal and lysosomal compartments and fuse with the endogenous mitochondrial network, while some remain associated with lysosomes and are degraded through hydrolysis and subsequent phagocytosis.

Extended Data Figure 6 | Tables containing the raw data used for quantitation of mitochondrial colocalisation with early endosomes, late endosomes, lysosomes, and endogenous mitochondria at 0.5, 1, 2, and 4 h. Values represent enumerated cardiomyocytes and exogenous mitochondria unassociated with, encapsulated within, or escaping from each compartment in those cells along with the total exogenous mitochondria

analysed (left to right). Columns represent distinct high-power microscopic evaluations of prepared slides and totals are presented (bottom).

Movie S1 | Internalised exogenous HCF mitochondrial position within cardiomyocytes. An exogenous mitochondria (green) associated with the endogenous mitochondrial network (red) after 1 h in iPS-CMs. After fixation, cells were stained for the contractile apparatus with ACTN (white) and for nuclei with DAPI (blue). The movie is representative of four separate experiments and corresponds to the image displayed in Extended Data Figure 2a.

Movie S2 | Exogenous mitochondria encapsulated in cardiomyocyte late endosomes. Late endosomes (red) associated with GFP-labelled exogenous mitochondria (green) were imaged by 3-D SR-SIM. Examples of encapsulated and escaping mitochondria are shown after 1 h of incubation of isolated mitochondria and iPS-CMs. The movie is representative of four separate experiments and corresponds to the image displayed in Figure 2c.

Movie S3 | Fusion of exogenous HCF mitochondria with cardiomyocyte mitochondria. iPS-derived cardiomyocytes with RFP-labelled mitochondria (red) were treated for 4 h with isolated GFP-labelled mitochondria (green). After fixation, nuclei were stained with DAPI (blue) and slides were imaged using 3-D SR-SIM. The movie is representative of four separate experiments and corresponds to the image displayed in Figure 4a.

Movie S4 | Association of MFN1 with exogenous mitochondria fusing with cardiomyocyte mitochondria. 3-D SR-SIM of human cardiomyocytes containing RFP-labelled mitochondria (red) incubated with isolated GFP-labelled exogenous mitochondria (green) for 30 min. Coverslips were fixed and stained with a mitofusin-1 (MFN1) antibody (white). The movie is representative of four experiments and corresponds to the image displayed in Figure 4c.

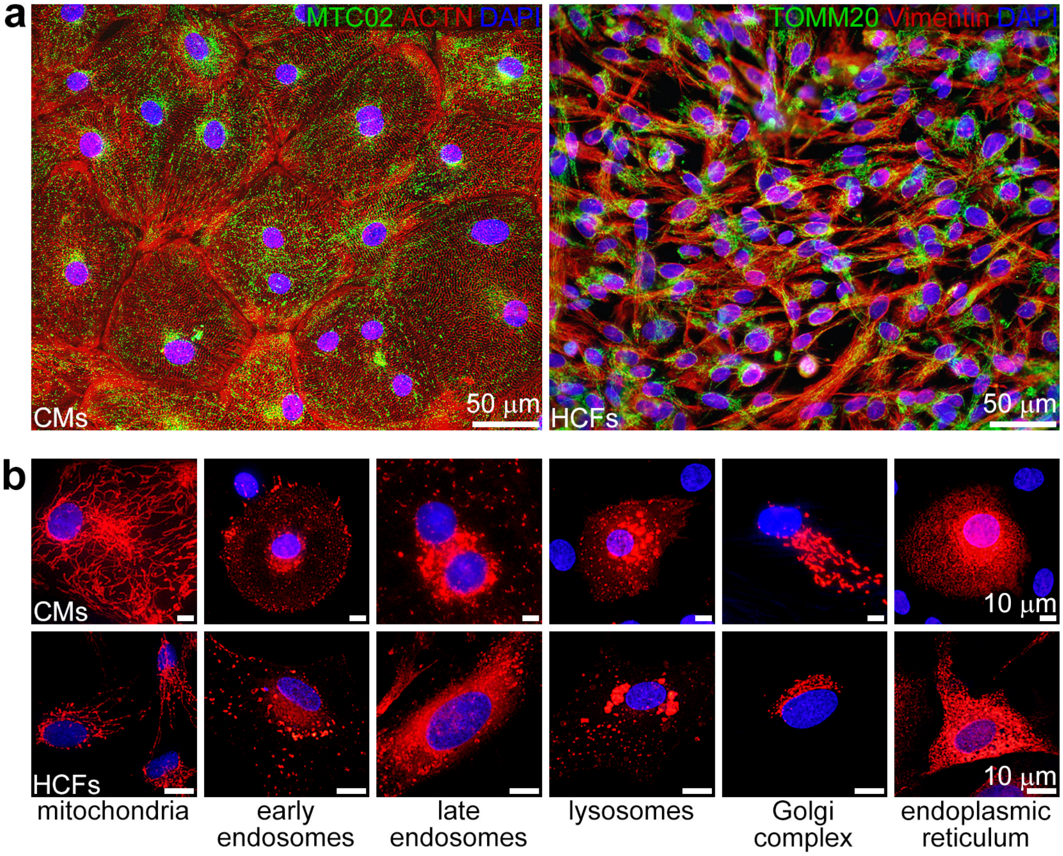
a
MTC02 ACTN DAPI
CMs
50 μm
TOMM20 Vimentin DAPI
HCFs
50 μm
b
CMs
10 μm
HCFs
10 μm
mitochondria
early endosomes
late endosomes
lysosomes
Golgi complex
endoplasmic reticulum

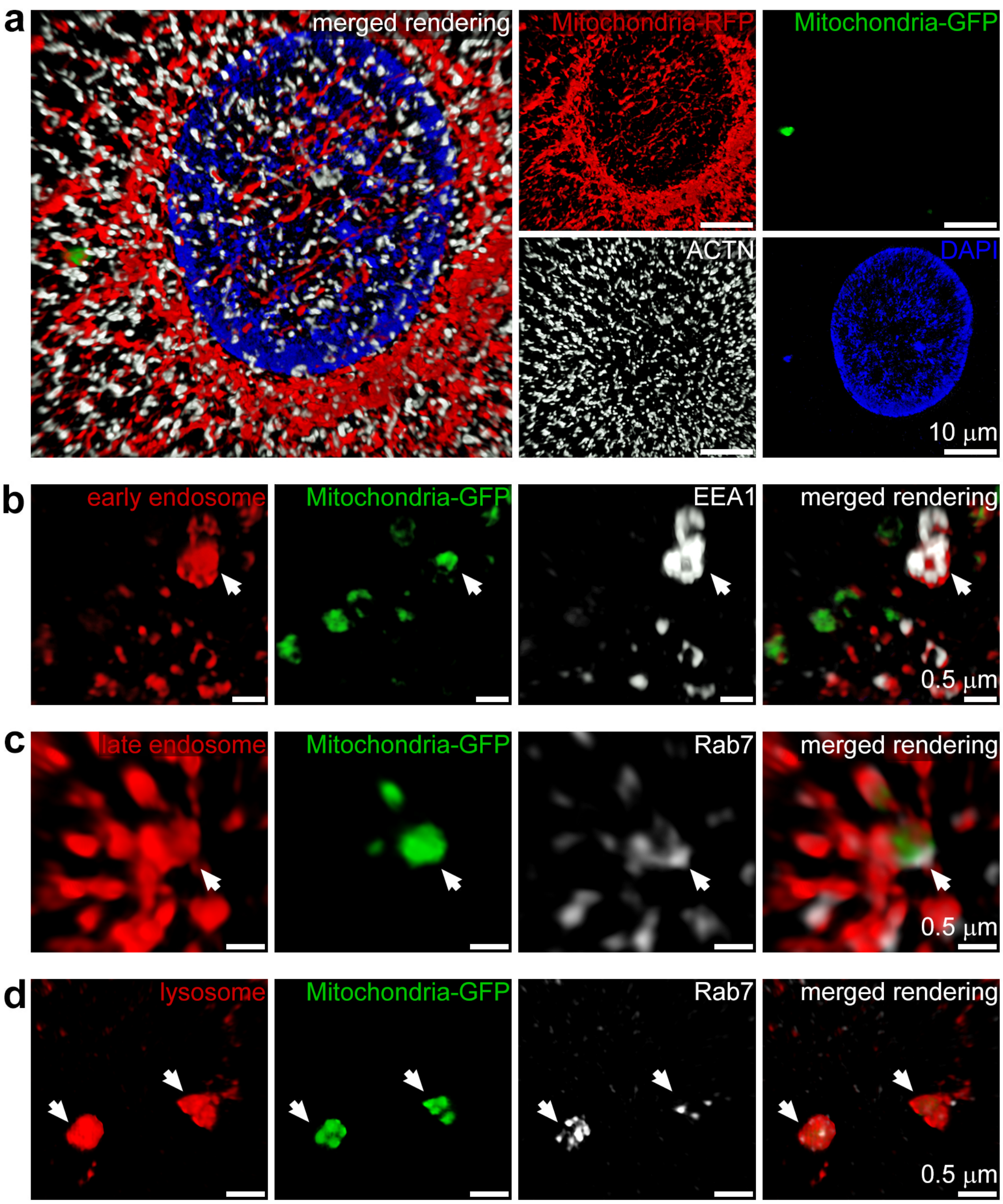
a
merged rendering
Mitochondria-RFP
Mitochondria-GFP
ACTN
DAPI
10 μm
b
early endosome
Mitochondria-GFP
EEA1
merged rendering
0.5 μm
c
late endosome
Mitochondria-GFP
Rab7
merged rendering
0.5 μm
d
lysosome
Mitochondria-GFP
Rab7
merged rendering
0.5 μm

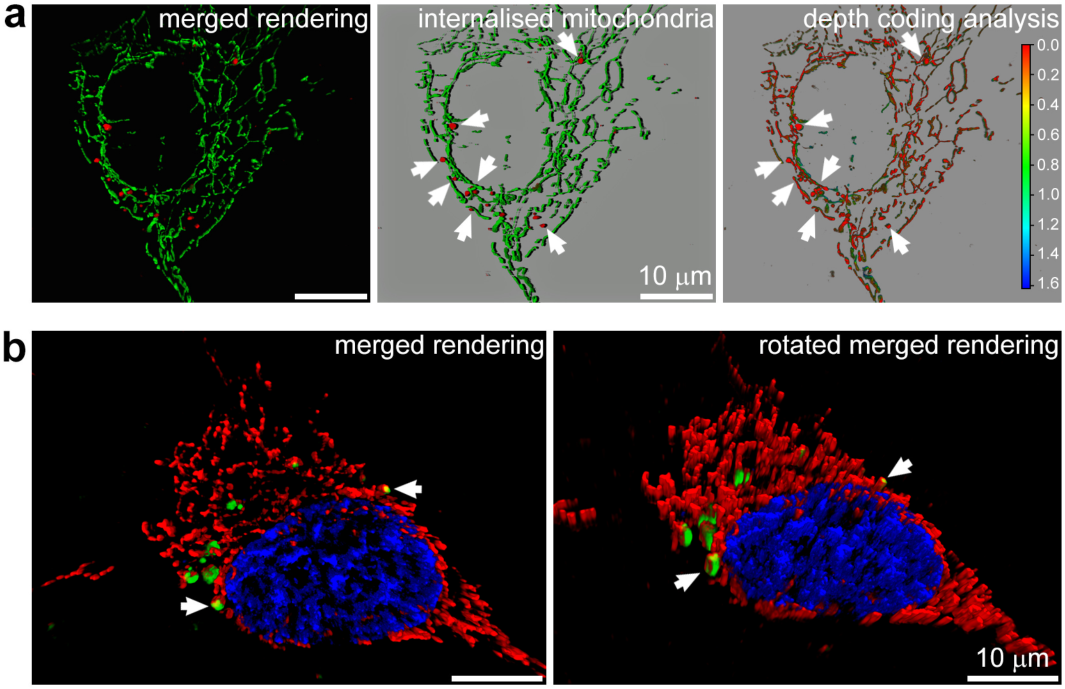
a
merged rendering
internalised mitochondria
10 μm
depth coding analysis
0.0
0.2
0.4
0.6
0.8
1.0
1.2
1.4
1.6
b
merged rendering
rotated merged rendering
10 μm

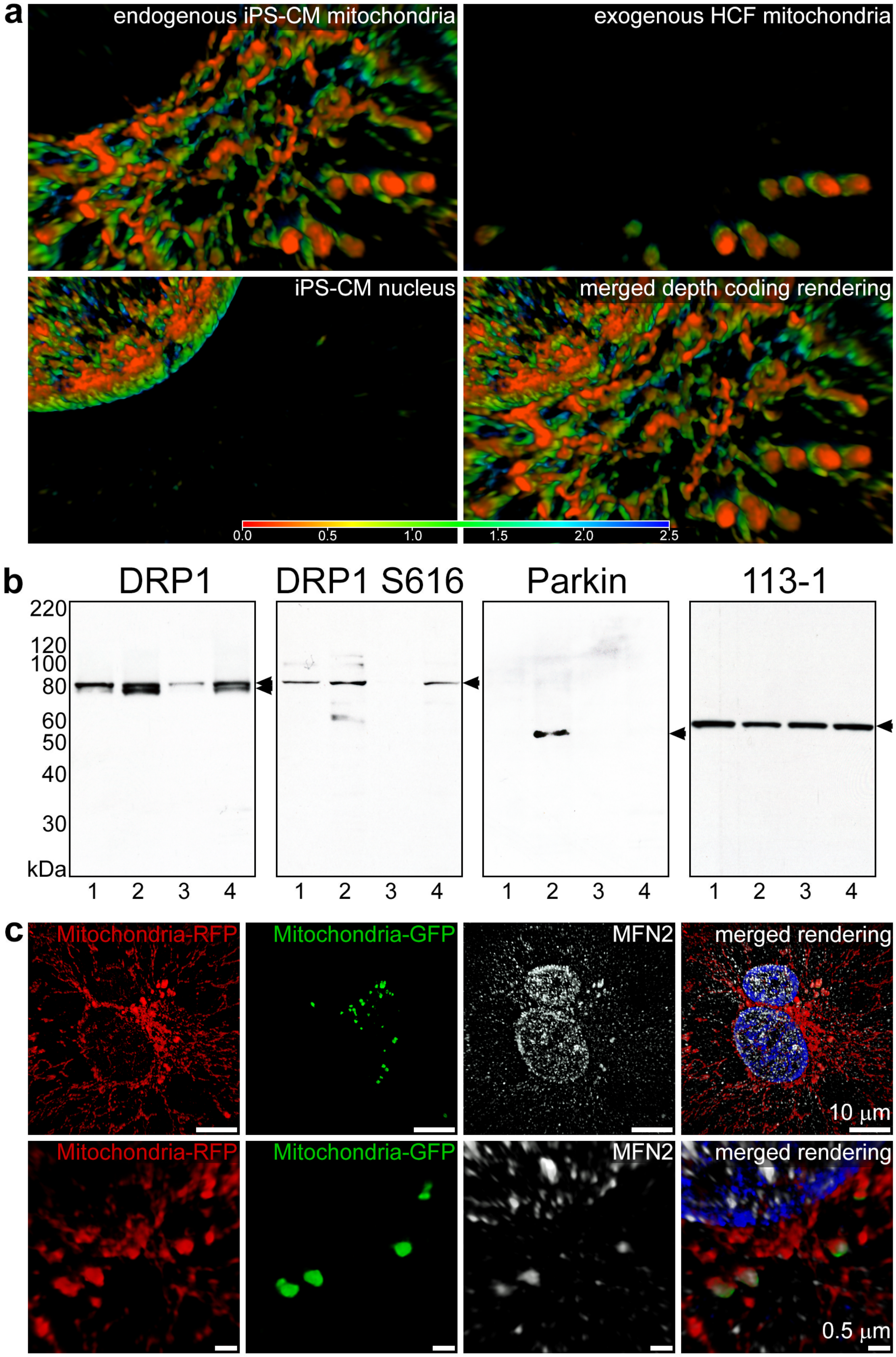
a
endogenous iPS-CM mitochondria
exogenous HCF mitochondria
iPS-CM nucleus
merged depth coding rendering
0.0
0.5
1.0
1.5
2.0
2.5
b
DRP1
DRP1 S616
Parkin
113-1
220
120
100
80
60
50
40
30
kDa
1 2 3 4
1 2 3 4
1 2 3 4
1 2 3 4
c
Mitochondria-RFP
Mitochondria-GFP
MFN2
merged rendering
10 μm
Mitochondria-RFP
Mitochondria-GFP
MFN2
merged rendering
0.5 μm

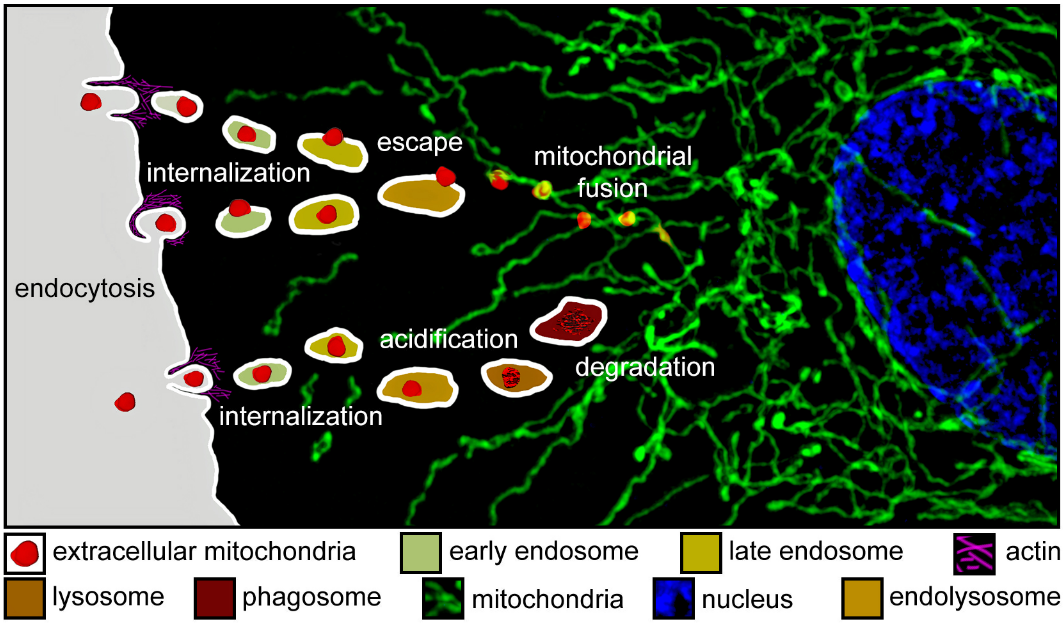

internalization
escape
mitochondrial fusion
endocytosis
acidification
degradation
internalization
extracellular mitochondria
early endosome
late endosome
actin
lysosome
phagosome
mitochondria
nucleus
endolysosome

Early Endosome - 0.5 hours

# cells	unassociated	encapsulated	escaping	total
12	7	249	50	306
14	6	161	46	213
13	15	212	69	296
15	9	371	58	438
22	43	242	68	353
14	9	138	35	182
9	11	128	23	162
13	13	213	45	271
10	16	648	65	729
6	5	333	34	372
16	13	436	54	503
15	30	648	64	742
10	1	451	50	502
10	2	556	57	615
179	180	4786	718	5684

Late Endosome - 0.5 hours

# cells	unassociated	encapsulated	escaping	total
29	3	163	63	229
14	8	222	52	282
20	6	232	51	289
20	15	113	62	190
17	10	157	33	200
15	15	214	64	293
20	16	165	47	228
21	4	255	44	303
24	3	102	12	117
21	8	187	28	223
23	9	170	42	221
23	5	282	36	323
26	9	233	56	298
23	2	242	55	299
21	2	171	39	212
23	7	272	36	315
21	7	229	33	269
15	15	259	49	323
13	6	201	40	247
17	8	183	39	230
12	4	227	32	263
9	2	141	11	154
13	0	220	27	247
19	2	192	36	230
14	6	231	31	268
473	172	5063	1018	6253

Lysosome - 0.5 hours

# cells	unassociated	encapsulated	escaping	total
16	2	68	74	144
10	4	134	7	145
11	1	145	21	167
13	2	134	79	215
18	5	135	31	171
10	2	145	29	176
14	2	148	32	182
10	0	64	23	87
11	0	62	16	78
5	0	57	3	60
10	0	84	14	98
7	0	159	4	163
12	2	79	16	97
10	3	144	10	157
14	5	331	47	383
6	3	127	13	143
8	3	112	13	128
7	3	267	3	273
19	3	493	25	521
10	9	264	25	298
11	5	426	15	446
9	0	164	12	176
8	1	60	4	65
10	1	261	23	285
10	8	117	19	144
269	64	4180	558	4802

Mitochondria - 0.5 hours

# cells	unassociated	associated	total
8	33	67	100
6	10	30	40
8	27	37	64
9	36	35	71
6	27	59	86
6	17	61	78
9	28	54	82
9	21	56	77
9	14	72	86
6	26	49	75
8	12	56	68
5	16	40	56
6	19	71	90
9	10	35	45
10	22	66	88
6	10	49	59
9	20	46	66
8	18	33	51
8	45	100	145
7	11	78	89
4	3	40	43
5	10	33	43
7	8	74	82
5	14	54	68
8	33	67	100
181	490	1362	1852

Early Endosome - 1 hour

# cells	unassociated	encapsulated	escaping	total
7	22	196	49	267
11	72	586	26	684
15	68	402	104	574
14	103	441	102	646
11	41	41	29	111
9	36	195	55	286
7	64	195	67	326
7	54	160	26	240
9	31	210	40	281
6	36	82	20	138
4	41	150	39	230
100	568	2658	557	3783

Late Endosome - 1 hour

# cells	unassociated	encapsulated	escaping	total
10	2	49	11	62
10	10	65	35	110
17	16	57	33	106
9	11	75	26	112
7	5	49	21	75
11	12	92	66	170
10	9	86	24	119
11	9	81	33	123
10	11	73	18	102
15	7	132	41	180
8	11	67	30	108
12	17	92	42	151
9	9	27	13	49
11	11	46	17	74
15	14	112	18	144
8	6	70	7	83
16	8	111	30	149
17	10	108	22	140
11	5	66	11	82
13	17	126	32	175
18	3	119	22	144
8	9	48	11	68
14	12	102	25	139
12	10	61	26	97
16	14	94	20	128
298	248	2008	634	2890

Lysosome - 1 hour

# cells	unassociated	encapsulated	escaping	total
9	12	117	13	142
11	15	180	28	223
10	10	113	33	156
10	3	185	24	212
12	9	147	73	229
10	7	170	98	275
10	5	108	26	139
8	2	158	15	175
10	3	143	41	187
12	5	112	53	170
11	4	207	48	259
7	10	143	33	186
9	2	143	28	173
9	1	135	38	174
11	3	214	48	265
12	5	186	38	229
16	1	223	66	290
12	1	109	42	152
8	4	79	22	105
8	0	121	39	160
10	15	168	112	295
9	4	121	31	156
12	3	93	34	130
8	2	175	27	204
9	8	147	48	203
253	134	3697	1058	4889

Mitochondria - 1 hour

# cells	unassociated	associated	total
10	32	23	55
9	56	35	91
10	49	48	97
10	33	65	98
11	38	64	102
10	111	77	188
5	19	56	75
9	51	80	131
8	53	82	135
9	62	81	143
10	34	34	68
14	27	127	154
8	44	81	125
12	36	67	103
7	21	35	56
8	20	64	84
10	32	48	80
12	22	48	70
10	30	45	75
9	12	46	58
9	31	64	95
8	29	35	64
12	28	58	86
10	24	68	92
10	64	83	147
240	958	1514	2472

Early Endosome - 2 hours

# cells	unassociated	encapsulated	escaping	total
11	75	366	174	615
19	143	336	114	593
10	117	422	92	631
7	20	186	42	248
16	157	453	128	738
13	162	189	64	415
11	45	308	82	435
11	119	513	201	833
6	43	264	80	387
6	29	249	55	333
7	41	439	48	528
6	46	100	49	195
16	219	389	131	739
11	109	452	133	694
7	43	147	47	237
7	86	179	70	335
17	263	163	92	518
20	221	256	173	650
201	1938	5411	1775	9124

Late Endosome - 2 hours

# cells	unassociated	encapsulated	escaping	total
10	12	117	39	168
9	9	161	70	240
10	9	118	59	186
13	4	222	40	266
9	2	114	43	159
14	0	189	33	222
16	2	209	21	232
17	0	137	43	180
8	1	89	32	122
9	1	150	44	195
12	3	117	27	147
16	3	153	69	225
20	5	230	59	294
8	3	112	31	146
10	10	78	39	127
7	7	97	7	111
10	3	74	23	100
10	5	121	32	158
9	1	173	56	230
11	5	188	50	243
10	1	86	26	113
8	2	87	20	109
10	4	175	23	202
6	0	103	13	116
6	5	106	25	136
268	97	3406	924	4427

Lysosome - 2 hours

# cells	unassociated	encapsulated	escaping	total
10	1	76	24	101
8	0	36	9	45
14	4	88	29	121
9	1	70	16	87
7	1	46	15	62
8	0	73	12	85
13	0	67	10	77
8	0	127	26	153
8	1	80	23	104
9	2	192	18	212
6	0	49	4	53
7	0	52	24	76
12	3	71	8	82
9	2	66	17	85
8	0	91	36	127
11	7	61	25	93
8	3	45	16	64
10	1	67	6	74
10	7	48	38	93
9	5	63	23	91
10	0	43	10	53
8	0	68	8	76
11	1	51	7	59
8	7	39	16	62
9	4	58	10	72
230	50	1727	430	2207

Mitochondria - 2 hours

# cells	unassociated	associated	total
11	9	135	144
5	1	68	69
6	5	66	71
7	6	67	73
8	5	68	73
12	12	132	144
8	7	72	79
6	14	74	88
8	6	123	129
6	8	80	88
9	8	95	103
12	1	58	59
7	3	50	53
8	2	67	69
13	9	134	143
7	9	41	50
11	15	114	129
7	3	44	47
8	3	63	66
8	9	93	102
3	0	50	50
5	8	57	65
10	11	109	120
11	11	76	87
8	9	62	71
204	174	1998	2172

Early Endosome - 4 hours

# cells	unassociated	encapsulated	escaping	total
10	163	218	53	434
9	182	134	53	369
11	202	126	46	374
9	328	333	61	722
12	255	171	61	487
10	102	137	33	272
11	203	316	58	577
13	194	243	75	512
9	142	182	28	352
12	287	214	38	539
8	198	215	37	450
8	67	69	36	172
13	322	318	88	728
9	169	182	52	403
16	322	354	71	747
5	193	50	30	273
12	220	197	61	478
10	251	165	55	471
12	347	357	69	773
12	174	182	54	410
14	305	247	71	623
8	99	183	41	323
12	215	308	65	588
13	270	304	61	635
11	160	91	44	295
269	5370	5296	1341	12007

Late Endosome - 4 hours

# cells	unassociated	encapsulated	escaping	total
8	81	96	16	193
12	151	110	65	326
13	137	149	105	391
9	172	52	49	273
13	141	91	38	270
13	141	93	46	280
11	92	78	67	237
15	94	114	83	291
10	144	63	41	248
13	120	56	51	227
13	133	78	67	278
13	62	58	70	190
17	71	116	117	304
12	48	87	49	184
10	50	72	21	143
12	90	61	63	214
10	82	53	26	161
18	130	84	102	316
13	157	77	80	314
7	126	62	59	247
11	200	38	39	277
11	133	78	91	302
12	93	100	97	290
6	71	69	39	179
19	138	95	94	327
301	2857	2030	1575	6462

Lysosome - 4 hours

# cells	unassociated	encapsulated	escaping	total
5	21	6	3	30
6	15	36	27	78
4	27	17	7	51
9	30	34	17	81
9	35	27	42	104
12	27	51	26	104
7	46	35	15	96
9	32	47	26	105
12	49	74	51	174
9	18	37	12	67
7	54	20	19	93
6	43	16	13	72
8	135	18	59	212
8	102	47	73	222
8	94	38	61	193
7	135	33	64	232
10	75	57	60	192
7	85	53	52	190
10	91	62	48	201
7	109	57	64	230
12	137	59	79	275
9	127	50	67	244
8	43	11	29	83
8	193	34	50	277
8	107	11	40	158
205	1830	930	1004	3764

Mitochondria - 4 hours

# cells	unassociated	associated	total
13	53	131	184
14	59	168	227
11	49	100	149
8	65	83	148
10	37	69	106
12	52	176	228
11	53	104	157
11	67	167	234
14	62	162	224
13	112	202	314
9	35	40	75
9	78	58	136
10	91	82	173
11	79	52	131
10	85	70	155
9	42	36	78
8	47	55	102
9	38	44	82
11	37	88	125
7	30	35	65
9	63	76	139
11	68	79	147
11	80	132	212
9	31	66	97
14	47	58	105
264	1460	2333	3793
